# Centralized repeated resectability assessment of patients with colorectal liver metastases during first-line treatment: prospective study

**DOI:** 10.1093/bjs/znaa145

**Published:** 2021-03-22

**Authors:** H Isoniemi, A Uutela, A Nordin, E Lantto, I Kellokumpu, A Ovissi, J Kosunen, R Kallio, L M Soveri, T Salminen, A Ålgars, A Lamminmäki, P Halonen, R Ristamäki, J Räsänen, H Karjula, Y Vaalavuo, M Lavonius, P Osterlund

**Affiliations:** 1 Department of Transplantation and Liver Surgery, Helsinki University Hospital and University of Helsinki, Helsinki, Finland; 2 Department of Radiology, Helsinki University Hospital, Päijät-Häme Central Hospital, Lahti, Finland; 3 Department of Surgery, Central Hospital of Central Finland, Jyväskylä, Finland; 4 Department of Radiology, Helsinki University Hospital and University of Helsinki, Helsinki, Finland; 5 Department of Oncology, Oulu University Hospital, Oulu, Finland; 6 Department of Oncology, Helsinki University Hospital, Helsinki, Finland; 7 Hyvinkää Hospital and Home Care, Hyvinkää, Finland; 8 Department of Oncology, Tampere University Hospital and University of Tampere, Tampere, Finland; 9 Department of Oncology, Turku University Hospital and University of Turku, Turku, Finland; 10 Department of Oncology, Kuopio University Hospital, Kuopio, Finland; 11 Department of Thoracic Surgery, Helsinki University Hospital and University of Helsinki, Helsinki, Finland; 12 Department of Surgery, Oulu University Hospital, Oulu, Finland; 13 Department of Surgery, Tampere University Hospital and University of Tampere, Tampere, Finland; 14 Department of Surgery, Turku University Hospital and University of Turku, Turku, Finland

## Abstract

**Background:**

Metastasectomy is probably underused in metastatic colorectal cancer. The aim of this study was to investigate the effect of centralized repeated assessment on resectability rate of liver metastases.

**Methods:**

The prospective RAXO study was a nationwide study in Finland. Patients with treatable metastatic colorectal cancer at any site were eligible. This planned substudy included patients with baseline liver metastases between 2012 and 2018. Resectability was reassessed by the multidisciplinary team at Helsinki tertiary referral centre upfront and twice during first-line systemic therapy. Outcomes were resectability rates, management changes, and survival.

**Results:**

Of 812 patients included, 301 (37.1 per cent) had liver-only metastases. Of these, tumours were categorized as upfront resectable in 161 (53.5 per cent), and became amenable to surgery during systemic treatment in 63 (20.9 per cent). Some 207 patients (68.7 per cent) eventually underwent liver resection or ablation. At baseline, a discrepancy in resectability between central and local judgement was noted for 102 patients (33.9 per cent). Median disease-free survival (DFS) after first resection was 20 months and overall survival (OS) 79 months. Median OS after diagnosis of metastatic colorectal cancer was 80, 32, and 21 months in R0–1 resection, R2/ablation, and non-resected groups, and 5-year OS rates were 68, 37, and 9 per cent, respectively. Liver and extrahepatic metastases were present in 511 patients. Of these, tumours in 72 patients (14.1 per cent) were categorized as upfront resectable, and 53 patients (10.4 per cent) became eligible for surgery. Eventually 110 patients (21.5 per cent) underwent liver resection or ablation. At baseline, a discrepancy between local and central resectability was noted for 116 patients (22.7 per cent). Median DFS from first resection was 7 months and median OS 55 months. Median OS after diagnosis of metastatic colorectal cancer was 79, 42, and 17 months in R0–1 resection, R2/ablation, and non-resected groups, with 5-year OS rates of 65, 39, and 2 per cent, respectively.

**Conclusion:**

Repeated centralized resectability assessment in patients with colorectal liver metastases improved resection and survival rates.

## Teaser

Repeated centralized assessment of resectability of liver and extrahepatic metastases in metastatic colorectal cancer yields high resectability and conversion rates. Patients undergoing curative resection of metastases limited to the liver had a 5-year overall survival rate of 68 per cent from diagnosis, whereas the rate among those with liver and extrahepatic metastases was 65 per cent.

Central revision increased resection rates

## Resumen

**Antecedentes:**

Es probable que la metastasectomía esté infrautilizada en el cáncer colorrectal metastásico (*metastatic colorectal cáncer*, mCRC). El objetivo de este estudio fue investigar el efecto que pudiera tener la evaluación centralizada periódica en la tasa de resecabilidad de las metástasis hepáticas.

**Métodos:**

El estudio prospectivo RAXO fue un estudio nacional finlandés. Se incluyó cualquier paciente con mCRC susceptible de tratamiento independientemente de su ubicación geográfica. Este subestudio incluyó los pacientes con metástasis hepáticas en el momento del diagnóstico entre 2012 y 2018. Se evaluó la resecabilidad por parte del comité multidisciplinar del centro de referencia terciario de Helsinki en el momento del diagnóstico y dos veces más durante el tratamiento sistémico de primera línea. Se valoró la tasa de resecabilidad, los cambios en el tratamiento y la supervivencia.

**Resultados:**

De los 812 pacientes incluidos, 301 (37%) tenían únicamente metástasis hepáticas. De estos, 161 (53%) se clasificaron inicialmente como resecables y 63 (21%) fueron rescatados durante el tratamiento sistémico. En 207 (69%) pacientes se realizó finalmente una resección o una ablación hepática. Al inicio del estudio, hubo discrepancia en cuanto a la resecabilidad entre el centro terciario y el local en 102 (34%) pacientes. La mediana de supervivencia libre de enfermedad (*median disease-free survival*, mDFS) desde la primera resección fue de 20 meses y la mediana de supervivencia global (*median overall survival*, mOS) de 79 meses. La mOS desde el diagnóstico de mCCR fue de 80, 32, 21 meses para los R0/1 resecados, los R2/ablacionados y los no resecados y la mOS a los 5 años fue del 68%, 37%, 9%, respectivamente. Se detectaron metástasis hepáticas y extrahepáticas en 511 pacientes. De estos, 72 (14%) pacientes se clasificaron inicialmente como resecables y 53 (11%) se rescataron. Finalmente, 110 (22%) pacientes se sometieron a resección o ablación hepática. En condiciones basales, había discrepancias en la resecabilidad entre el centro terciario y el equipo local en 116 (23%) pacientes. La mDFS desde la primera resección fue de 7 meses y la mOS de 55 meses. La mOS desde el diagnóstico de mCCR fue de 79, 42, 17 meses en los R0/1 resecados, R2/ablacionados y los no resecados, y la OS a 5 años fue del 65%, 39%, 2%, respectivamente.

**Conclusión:**

La evaluación centralizada y periódica de la resecabilidad en pacientes con metástasis hepáticas colorrectales mejoró las tasas de resección y supervivencia.

## Introduction

Hepatic resection is considered potentially curative for patients with colorectal liver metastases (CRLMs), with 5-year overall survival (OS) rates of 30–50 per cent^1^. Resectability rates have been increasing as a result of improved surgical techniques and conversion therapy with chemotherapy and targeted agents^2–6^. There is a strong correlation between response to treatment and resection rate^4^. In studies that enrolled selected patients with liver-only metastases, 24–54 per cent of patients underwent tumour resection after systemic therapy, compared with 1–26 per cent of patients in studies that included unselected patients with CRLMs^4^.

Resectability can also be improved by interventional methods, such as local ablative therapy (LAT)^7–9^. Technical resectability has become a reality in more demanding surgery for liver metastases. Good results have been shown even in patients with 10 or more liver metastases, with two-stage hepatectomy for bilobar metastases and after second-line conversion therapy^10–12^.

Resectability is generally defined based on the ability to remove all metastases with clear margins while maintaining sufficient future liver remnant. If extrahepatic metastases are present, all sites should be amenable to curative treatment. The decision regarding liver resectability is easy if only a few small metastases are present without involvement of crucial structures, such as central vessels or bile ducts, or if the liver is full of metastases, but borderline resectable disease is more challenging.

Multidisciplinary teams (MDTs) are increasingly being engaged in assessment and improvement of resectability^13^. Decisions between palliative care or treatment with curative intent can be difficult, and opinions may differ, depending on the experience and expertise of the MDT. A study^2^ assessing borderline resectability revealed great variability in views on resectability among liver surgeons both upfront and after conversion therapy. A recent study^14^ showed significant variation in opinions between specialties, with frequent underestimation of resectability by medical oncologists and non-hepatobiliary surgeons.

The hypothesis is that resection is probably underused in real-world oncology practice. The aim of this study was to assess real-world resectability and conversion rates and survival in patients fit for oncological treatment of CRLMs with centralized repeated assessment.

## Methods

This was a planned substudy of patients with baseline liver metastases with or without extrahepatic metastatic sites, from the prospective nationwide investigator-initiated RAXO study (NCT01531621, EudraCT2011-003158-24). Treatable patients, defined as those with metastatic colorectal cancer fit for oncological treatment, referred to oncology units were recruited. Inclusion criteria were: histologically confirmed colorectal cancer with liver metastases at baseline; patient scheduled for first-line systemic therapy; age over 18 years; and signed written informed consent obtained according to Good Clinical Practice (GCP). The ethics committee at Helsinki University Hospital (242/13/03/02/2011) and each hospital approved the study. The primary objective was to assess the overall resectability and conversion of metastases, and outcomes after resection. The protocol is available from the authors on request.

### Protocol for assessing resectability

CRLMs were considered resectable if complete resection with tumour-free margins was feasible, and at least 30 per cent of liver volume would be preserved, including at least two Couinaud’s segments with adequate vascular inflow and outflow and biliary drainage. All sites should be resectable in patients with extrahepatic metastases. All metastatic sites were recorded from baseline until death or end of follow-up, and potential resectability was assessed.

Resectability was recorded prospectively and centralized at Helsinki University Hospital, which is a tertiary liver centre performing over 200 liver resections and 60–70 liver transplant annually. The oncologist submitted information on the primary tumour, TNM stage, metastatic sites, and dates of primary diagnosis and metastatic colorectal cancer to a secure online database (https://www.raxo.fi) (*Fig. S1*). All available imaging studies were provided to the tertiary centre for a second opinion. The MDT consisted of experienced liver surgeons, abdominal radiologists, and other specialists, such as gastrointestinal surgeons, thoracic surgeons, cytoreductive surgeons, gynaecologists, thoracic radiologists, and PET–CT specialists as needed. The resectability of liver and other metastases was assessed at time of diagnosis of metastatic colorectal cancer, and at the first and second response evaluation (at 8–10-week intervals) during first-line therapy.

Local assessment of baseline resectability outside the tertiary referral centre was recorded as upfront resectable with or without neoadjuvant therapy, borderline starting conversion therapy, or non-resectable.

### Imaging

CT of the chest, abdomen, and pelvis was used to image metastases. MRI was added in hepatic steatosis or if CT was not unequivocal. [^18^F]fluorodeoxyglucose PET–CT was used in selected patients to evaluate the extrahepatic spread if CT was not conclusive or there was a clinical discrepancy.

Guidelines for CT protocols were standardized in all hospitals. Patients underwent scanning with 64-/128-slice CT after administration of low-osmolarity non-ionic contrast (iodine concentration 350 mg/ml at 3 ml/s). Baseline CT included the chest and upper abdomen in the late arterial phase, and the abdomen and pelvis in the portal venous phase. Follow-up CT was performed during the portal venous phase. CT images were reconstructed with 3-mm slices.

### Second opinion on resectability

The second opinion was provided online to the database (https://www.raxo.fi) using a structured form on which number of CRLMs, size of the largest lesion, location (unilobar/bilobar), and affected liver segments were recorded (*Fig. S2*). The statement of resectability included three options: resectable, borderline resectable but possibly convertible, and non-resectable. The five reasons for non-resectability were location, number of metastases (denoted as 15, if more than 15), size of the largest lesion, non-treatable extrahepatic spread, or other. Interventions, such as resections and/or LAT, were performed according to clinical practice; liver resections were undertaken at six centres, with referral of those requiring demanding procedures to the tertiary unit.

The lungs were always assessed also. Number and size of metastases, and involved lobes were recorded, and denoted as resectable or non-resectable. Thoracic surgeons were consulted as needed. Other extrahepatic metastases were noted as resectable or not.

Helsinki tertiary centre and the five other hospitals performing liver resections retrospectively compared their CRLM resection rates during the prospective study period from 2012 to 2018, with the preceding period from 2005 to 2011. The Helsinki tertiary centre is responsible for liver MDTs and resections in the Helsinki University region and for seven other regional hospitals. The most demanding liver surgery (such as 2-stage procedures, most major hepatectomies) from any part of Finland is centralized to Helsinki according to state regulation. Finland is divided into five university hospital catchment areas and the resection rate per million population was compared between these areas.

### Systemic therapy

Each hospital used its own standard treatment protocols based on National Comprehensive Cancer Network and European Society for Medical Oncology guidelines. Systemic therapy was given until disease progression or toxicity occurred, or resectability had been achieved. For upfront resectable metastatic disease, perioperative oxaliplatin-based treatment was preferred^15^. In the conversion setting, the most intensive regimen that could be tolerated was used, usually a doublet or triplet chemotherapy regimen combined with targeted agents, such as bevacizumab, cetuximab, or panitumumab, according to *RAS*/*BRAF* status and sidedness^7^. The recommendation was to continue the same chemotherapy for 3 months after resection at the physician’s discretion. Targeted agents were not generally used in the adjuvant setting after publication of negative cetuximab and bevacizumab findings^16–18^. Treatment with bevacizumab was stopped 5–6 weeks before surgical intervention. One additional cycle of chemotherapy was subsequently administered before surgical exploration if tolerated by the patient. Chemotherapy was restarted 4 weeks after surgery in the adjuvant setting if there were no complications that delayed its initiation.

### Follow-up

Patients were followed after resection according to the study protocol. First imaging with CT of the chest, abdomen, and pelvis, laboratory tests including tumour markers (carcinoembryonic antigen with or without carbohydrate antigen 19-9), and clinical evaluation were done 2–3 months after resection. Thereafter, patients were followed at 3-month intervals to 2 years and at 6-month intervals up to 5 years. Response evaluation was performed every 2–3 months for patients receiving systemic treatment.

### Statistical analysis

The main objectives according to the protocol were to analyse resectability, conversion, and median disease-free survival (DFS), progression-free survival (PFS), and OS. Survival was assessed by Kaplan–Meier survival analysis with log rank test, and Cox multivariable regression analysis. The cut-off date for survival status was 7 February 2020. Median OS was calculated from first intervention (resection or LAT) to death from any cause or censored at last follow-up. DFS was calculated from first intervention to relapse of metastatic colorectal cancer, death from any cause, or censored at last date of follow-up. PFS was calculated from metastatic colorectal cancer diagnosis to progression on systemic therapy, relapse after intervention, death from any cause, or censored at the last date of follow-up. OS after diagnosis of metastatic colorectal cancer was calculated until any cause of death or censoring at last follow-up. Two-sided *P* < 0.050 was considered statistically significant.

A sample size calculation was performed. To detect a hazard ratio (HR) for death of 0.70 in the resected and/or ablated group compared with the no-resection group treated with first-line systemic therapy, with a two-sided type I error of 0.05 and type II error of 0.20, 671 patients were needed, assuming median OS of 2 years in the no-resection group^19^. A 5-year accrual period was planned, with assumed 1 per cent loss to survival follow-up. Based on a presumed treatment allocation of 25 per cent resected or ablated and 75 per cent treated with systemic therapy, 329 deaths were targeted for the final OS analysis.

## Results

Between June 2012 and October 2018, a total of 1086 patients with metastatic colorectal cancer at all 21 oncology units in Finland were included (The RAXO study group is presented in *Appendix S1*). Some 812 patients with colorectal cancer and liver metastases were enrolled in this planned substudy, of whom 301 had metastatic colorectal cancer limited to the liver, and 511 had liver and extrahepatic metastases. Consent was obtained from approximately 40 per cent of eligible patients nationwide; the enrolment rate was 58 per cent at the five university hospitals and two largest regional hospitals with dedicated study personnel available, and 31 per cent at smaller regional hospitals where local oncologists did all study procedures. At inclusion, all patients were eligible for chemotherapy, but 20 (2 per cent) eventually received best supportive care only and were included in the systemic therapy group according to the intention-to-treat principle. At the data cut-off point, 71 per cent of patients had died. Median follow-up was 59 (range 17–88) months. Patient characteristics in the two groups are shown in *Table 1*.

**Table 1:** Demographics and baseline characteristics of patients with metastatic colorectal cancer with liver-only disease or liver metastases and extrahepatic sites

		**Liver-only metastases** **(*n* = 301)**	**Liver and extrahepatic metastases** **(*n* = 511)**
**Demographics**			
Age (years)*		66 (26–87)	66 (24–90)
	≤ 70	202 (67.1)	345 (67.5)
	≥ 70	99 (32.9)	166 (32.5)
Sex	M	196 (65.1)	316 (61.8)
	F	105 (34.9)	195 (38.2)
ECOG perormance status	0	112 (37.2)	107 (20.9)
	1	154 (51.2)	286 (56.0)
	2–3	35 (11.6)	118 (23.1)

**Primary tumour**			
Location	Right colon	79 (26.2)	140 (27.4)
	Left colon	154 (51.2)	168 (32.9)
	Rectum	67 (22.3)	201 (39.3)
	Multiple	1 (0.3)	2 (0.3)
Surgery			
	Upfront or simultaneous	301 (100)	203 (39.7)
	After neoadjuvant therapy/conversion	0 (0)	92 (18.0)
	Primary not operated	0 (0)	216 (42.3)

**Metastases**			
Presentation			
	Synchronous	178 (59.1)	436 (85.3)
	Metachronous	123 (40.9)	75 (14.7)
No. of sites	1	301 (100)	0 (0)
	2	0 (0)	254 (49.7)
	3–6	0 (0)	257 (50.3)
Liver	Bilateral	148 (49.2)	380 (74.4)
	Unilateral	153 (50.8)	131 (25.6)
	No. of lesions*	2 (1–15^†^)	6 (1–15^†^)
	≥ 15 lesions	35 (11.6)	183 (35.8)
	Largest lesion (mm)*	28 (5–190)	40 (5–200)
Lung	Baseline	0 (0)	218 (42.7)
	During follow-up	107 (35.5)	313 (61.3)
Peritoneal	Baseline	0 (0)	72 (14.1)
	During follow-up	36 (12.0)	127 (24.9)
Lymph nodes	Baseline	0 (0)	173 (33.9)
	During follow-up	75 (24.9)	249 (48.7)

**Patients who had R0–2 resection/LAT**		*n* = 207	*n* = 110
Interventions			
Liver^‡^	Major resection	99	50
	Minor resection	125	68
	LAT	41	16
Lung	Resection	23	40
Local relapse	Surgery	5	15
Gynaecological/urological	Resection	3	11
Peritoneal	Cytoreductive ± HIPEC	7	8
Lymph nodes	Lymphadenectomy	4	3
Skin/intramuscular	Excision	1	5

**Systemic therapy^§^**			
No. of lines	1	133 (44.2)	187 (36.6)
	2	74 (24.6)	125 (24.5)
	≥ 3	94 (31.2)	199 (38.9)
Chemotherapy	Fluoropyrimidine	289 (96.0)	490 (95.9)
	Oxaliplatin	194 (64.5)	307 (60.1)
	Irinotecan	80 (26.6)	132 (25.8)
VEGF inhibitor	Bevacizumab	144 (47.8)	310 (60.7)
	Aflibercept	3 (1.0)	3 (0.6)
EGFR inhibitor	Panitumumab	38 (12.6)	50 (9.8)
	Cetuximab	22 (7.3)	23 (4.5)

Values in parentheses are percentages unless indicated otherwise;

* values are median (range).

† Maximum number recorded was 15, even if more liver metastases were present.

‡ Total number of interventions (re-resections included); median 2 (range 1–10) per patient.

§ Maximum during all lines of therapy. ECOG, Eastern Cooperative Oncology Group; LAT, local ablative therapy; HIPEC, hyperthermic intraperitoneal chemotherapy; VEGF, vascular endothelial growth factor; EGFR, epidermal growth factor receptor.

### Resectability and resection rates

#### Patients with liver-only metastatic colorectal cancer

**Fig. 1:**
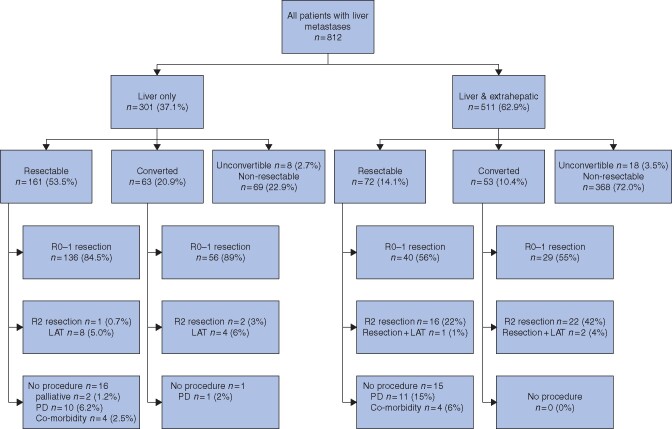
Study flow chart PD = progressive disease

**Fig. 2:**
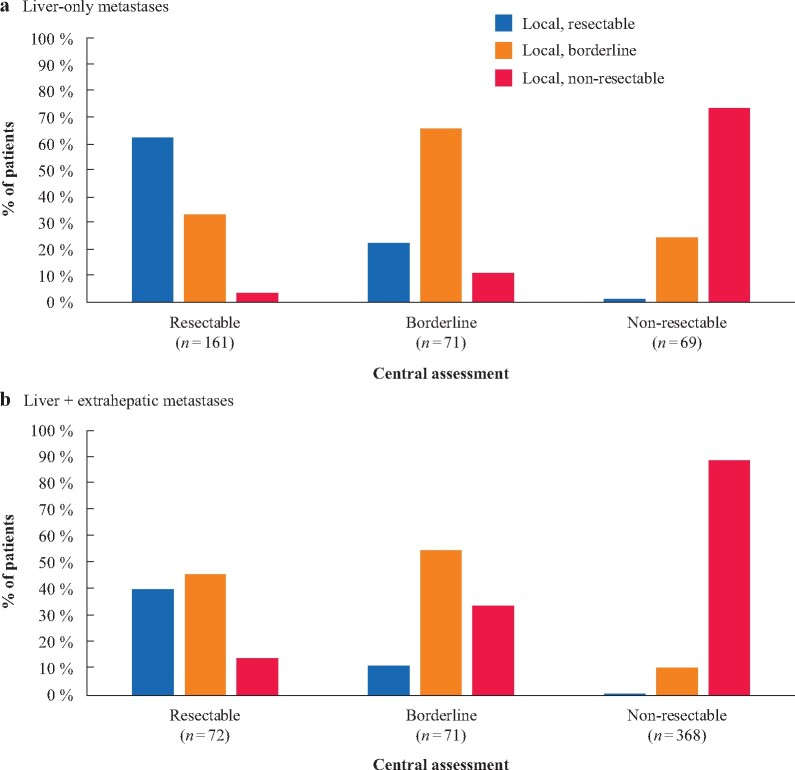
Rates of patients with upfront resectable, borderline, and non-resectable disease in central assessment compared local assessment **a** Liver-only and **b** liver and extrahepatic metastases.

At first central resectability evaluation, 161 of 301 patients with liver-only metastatic colorectal cancer (53.5 per cent) were considered to have upfront resectable tumours, whereas 71 (23.6 per cent) had borderline, and 69 (22.9 per cent) had non-resectable disease (*Fig. 1*). A discrepancy was noted between central and local resectability assessment at baseline in 102 patients (33.9 per cent) (*Fig. 2*). Local underestimation of resectability of tumours categorized centrally as resectable or borderline resectable was seen for 37.3 and 11.3 per cent of patients respectively. Local overestimation was observed in 22.5 and 26.1 per cent of patients considered to have borderline or unresectable disease at central re-evaluation. In the repeated resectability evaluation during conversion therapy, disease in 63 patients was converted (60 of 71 borderline and 3 of 69 non-resectable) (*Fig. 1*).

Of the 161 patients with disease that was resectable upfront, 145 (90.1 per cent) had an intervention (R0–1 resection, R2 resection or LAT) and 16 did not undergo tumour resection or ablation (*Fig. 1*). In total, a liver intervention was undertaken in 207 of 301 patients (68.8 per cent) and was curative in 192 (63.8 per cent). Tumour burden, new metastatic sites, and interventions are detailed in *Table 1* and *Table S1*. The liver or LAT rate was 41 (19.8 per cent) and median time to re-resection was 16 (95 per cent c.i. 11 to 21) months. Neoadjuvant, conversion, and/or adjuvant therapy was given to 94 and 63 per cent in conjunction with first and second resection respectively (*Table S1*).

#### Patients with concomitant liver and extrahepatic metastases

Extrahepatic metastases were present at 17 sites, most common pulmonary metastases, then distant lymph node metastases, peritoneal metastases, bone metastases, local sites, adrenal, ovarian, skin/subcutaneous, and/or brain metastases (*Table 1*). At first central resectability evaluation, 72 patients with liver and extrahepatic metastases (14.1 per cent) were considered to have resectable, 71 (13.9 per cent) borderline resectable, and 368 (72.0 per cent) non-resectable lesions (*Fig. 2*). A discrepancy was noted between baseline central and local resectability assessment in 116 of the 511 patients (22.7 per cent) (*Fig. 2*). Local underestimation of resectability for tumours categorized centrally as resectable or borderline was seen in 59.7 and 33.8 per cent of patients respectively. Local overestimation was noted in 11.3 and 11.1 per cent of patients who were considered centrally to have borderline resectable or unresectable disease respectively. On repeated evaluation during conversion therapy, tumours in 53 patients became eligible for surgery (*Fig. 1*).

Of 125 patients with tumours categorized as resectable either upfront or after conversion therapy, resection or ablation was performed in 110 (88.0 per cent), of whom 69 (55.2 per cent) underwent curative R0–1 resection (*Fig. 1*). Non-curative resections were more common among patients in the converted group (42 per cent) than in upfront resectable group (22 per cent) (*Fig. 1*), and were mostly due to progressive disease before second-site operation. Tumour burden, metastatic sites, and interventions are shown in *Table 1* and *Table S1*. Liver re-resection was performed in 18 patients (16.4 per cent) and median time to re-resection was 10 (95 per cent c.i. 9 to 12) months. Neoadjuvant, conversion, and/or adjuvant therapy was given to 91 and 73 per cent in conjunction with first and second resection (*Table S1*).

#### Comparison of resection numbers between two periods

The total number of CRLM resections at Helsinki tertiary centre (1.67 million inhabitants) increased from 260 in 2005–2011 to 472 during the study period (2012–2018), an 81.5 per cent increase. At seven referring hospitals (1.30 million inhabitants), the number of CRLM resections increased from 77 to 154 (100 per cent increase). In the five other hospitals performing liver resections (serving an area of 2.53 million inhabitants), numbers increased from 197 to 365 (85.3 per cent increase).

#### Comparison of resection rates in RAXO trial between university hospital catchment areas

The CRLM resection rate in the RAXO study was 144 per million population for Helsinki tertiary centre and 81–112 per million in the four other university regions. There were no differences in median OS after diagnosis of metastatic colorectal cancer among patients who underwent R0–1 resection between the five university catchment areas (HR 0.98, 95 per cent c.i. 0.85 to 1.13).

### Overall, disease-free, and progression-free survival

#### Liver-only metastatic colorectal cancer

**Fig. 3:**
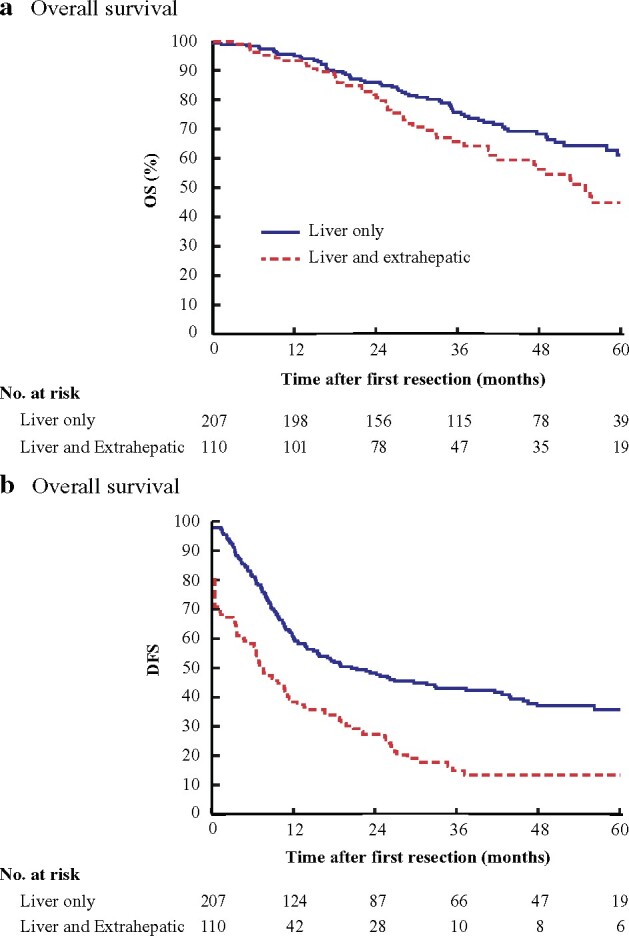
Overall and disease-free survival for patients with liver-only metastases or liver metastases with extrahepatic sites calculated from the time of first R0–2 resection and/or local ablative therapy **a** Overall survival and **b** Overall survival. **a** Hazard ratio (HR) 0.66 (95 per cent c.i. 0.46 to 0.95), *P* < 0.001; **b** HR 0.49 (0.38 to 0.65), *P* < 0.001 (Cox regression).

Median OS after the first resection or ablation in patients with liver-only metastatic colorectal cancer was 79 (95 per cent c.i. 63 to 96) months, and 3- and 5-year OS rates were 76 and 63 per cent respectively (*Fig. 3a*). Median DFS was 20 (11 to 30) months) (*Fig. 3b*).

**Fig. 4:**
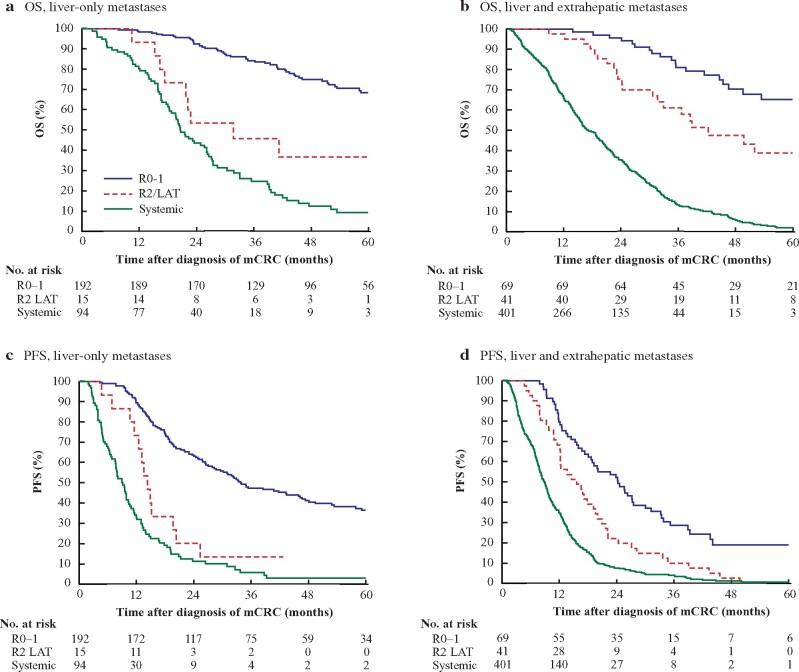
Overall survival after diagnosis of metastatic colorectal cancer and Progression free survival **a** Overall survival (OS) and **b** progression free survival (PFS) in patients with liver-only metastases, and **c** OS and **d** PFS in patients with liver and extrahepatic metastases according to treatment strategy. LAT, local ablative therapy; mCRC, metastatic colorectal cancer. **a** Hazard ratio (HR) 0.15 (95 per cent c.i. 0.10 to 0.21), *P* < 0.001 for R0–1; HR 0.70 (0.35 to 1.40), *P =* 0.304 for R2/LAT; **b** HR 0.19 (0.14 to 0.26), *P <* 0.001 for R0–1; HR 0.56 (0.32 to 0.98), *P =* 0.042 for R2/LAT; **c** HR 0.12 (0.08 to 0.19), *P <* 0.001 for R0–1; HR 0.33 (0.22 to 0.50), *P <* 0.001 for R2/LAT; **d** HR 0.28 (0.21 to 0.38), *P <* 0.001 for R0–1; HR 0.52 (0.38 to 0.73), *P <* 0.001 for R2/LAT; all *versus* systemic (log rank test).

Median OS after diagnosis of metastatic colorectal cancer in the R0–1 resection, R2/ LAT, and systemic therapy groups was (95 per cent c.i. 71–90), 32 (11 to 53), and 21 (17 to 24) months, with 5-year OS rates of 68, 37, and 9 per cent, respectively (*Fig. 4a*). OS calculated by the 12-month landmark method to control for guarantee-time bias is shown in *Fig. S3*. Median PFS was 34 (25 to 43), 14 (12 to 17), and 9 (7 to 11) months in the R0–R1 resection, R2/LAT, and systemic treatment groups respectively (*Fig. 4b*).

#### Liver and extrahepatic sites

Median OS after first resection/ablation in the liver and extrahepatic metastases group was 55 (95 per cent c.i. 47 to 64) months, with 3- and 5-year OS rates of 66 and 45 per cent respectively (*Fig. 3a*). Median DFS was 7 (4 to 9) months (*Fig. 3b*).

Median OS after diagnosis of metastatic colorectal cancer was 79 (61 to 97), 42 (17 to 57), and 17 (15 to 19) months in the R0–1 resection, R2/LAT, and systemic treatment groups, with 5-year OS rates of 65, 39, and 2 per cent, respectively (*Fig. 4c* and *Fig. S3*). Median PFS was 24 (18 to 31), 16 (10 to 21), and 9 (8 to 10) months respectively (*Fig. 4d*).

*Survival for all patients undergoing resection either upfront or after conversion*

**Fig. 5:**
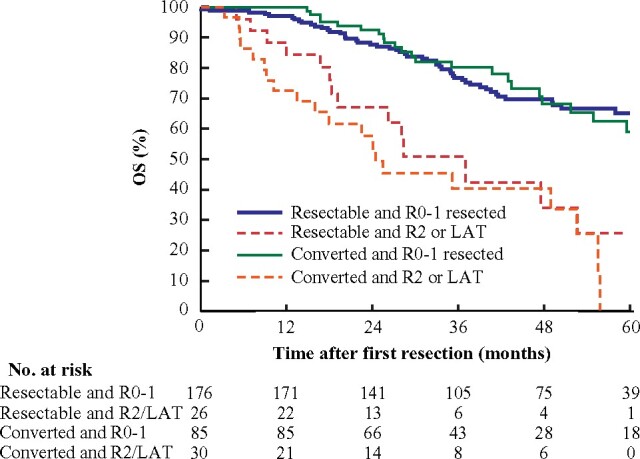
Overall survival after first resection in patients with upfront resectable or converted metastases according to type of resection Hazard ratio (HR) 0.23 (95 per cent c.i. 0.14 to 0.39), *P <* 0.001 for resectable and R0–1 resected; HR 0.22 (0.12 to 0.41), *P <* 0.001, for converted and R0–1 resected; and HR 0.74 (0.37 to 1.46), *P =* 0.385, resectable and R2 resected or local ablative therapy (LAT); all *versus* converted and R2/LAT (Cox regression).

OS after first resection among all patients who had curative resection did not differ between upfront resectable and converted subgroups (median 76 (95 per cent c.i. 71 to 80) months *versus* not reached) (*Fig. 5*). In the R2/LAT group, no difference was observed between upfront resected and converted subgroups. Median DFS was longer in the upfront resectable group than in the converted group (37 (95 per cent c.i. 22 to 52) *versus* 25 (11–39) months).

Five-year OS rates increased from 60 per cent for patients included in the first half of the study period (June 2012 to August 2015) to 68 per cent (September 2015 to October 2018) for patients with any intervention.

### Reasons for non-resectability

The main reasons for non-resectability of liver metastases evaluated upfront in 575 patients were: large number of metastases (192), poorly located metastases (117), size (45), co-morbidity (16), and non-resectable extrahepatic sites (205). Main reasons why patients who were considered to have resectable metastases did not undergo resection included: disease progression during neoadjuvant chemotherapy (22), co-morbidity (10), complete response to systemic therapy (4), and disease considered unresectable during surgical exploration (3).

## Discussion

This nationwide study showed the benefit of repeated central assessment of resectability of metastatic colorectal cancer. This pushed the boundaries for resectability, with high resection rates and encouraging survival. OS may increase significantly with use of repeated centralized resectability assessment at a tertiary referral centre.

In this study, the rate of rate of upfront resectability was 53.5 per cent among patients with liver-only metastases and 14.1 per cent in patients with concomitant extrahepatic disease, whereas lower rates have been reported in the literature for liver-only metastases^6^^,^^20–22^. The resection/ablation rate after conversion therapy for upfront borderline or non-resectable liver-only metastases was 45.0 per cent in this study, which is high compared with the 1–26 per cent in unselected population series, and in line with 24–61 per cent in selected series^4^^,^^5^ and a Dutch phase III study^23^ that assessed conversion prospectively. The intervention rate among patients who received conversion therapy was higher than in earlier studies^22^^,^^23^.

Discrepancy between local and central baseline assessment of resectability was common, underestimation being more common than overestimation. This was also observed among experienced liver surgeons^2^^,^^14^^,^^23^. Full information on the impact of second and third assessments on final resectability decisions taken locally is not available, but local decisions regarding why patients with resectable metastases or metastases that became resectable after conversion therapy did not undergo resection were recorded extensively.

The number of liver resections performed nationwide significantly increased over time. This is in line with recent population-based trends for increased liver resections^6^^,^^21^. Reasons include the effect of repeated central MDT assessment.

OS after resection or ablation of liver-only metastases was longer than in other series, for which 5-year survival rates ranged from 38 to 60 per cent depending on selection criteria^1^^,^^13^^,^^24–26^. The 5-year OS rate after liver resection for late metachronous metastases in a previous Finnish^20^ population-based study was 67 per cent. Patients with synchronous or early metachronous CRLMs had a reported OS rate of 44–46 per cent^20^^,^^21^.

Patients with extrahepatic disease who underwent resection or ablation had a longer survival than previously published^27^^,^^28^. Curative resection was difficult to achieve in this group, but resection was still worthwhile. Patients who did not undergo resection or ablation of liver metastases had a short survival, in line with previous findings^21^^,^^22^^,^^29^. If intervention was curative, survival was similar between the group of patients who had upfront resectable metastases and those who underwent liver resection after downsizing, in line with another study^30^. The same trend was noted in patients who underwent R2 resection or ablation.

The strengths of this prospective study include the use of repeated assessment of resectability according to the protocol, thorough recording of all treatments given during a long follow-up, and no patients lost to follow-up. One major limitation is that it is not population-based. Nationwide, approximately 40 per cent enrolment of patients with metastatic colorectal cancer was achieved, in line with the highest recruiting academic centres, and clearly higher than the general enrolment rate of less than 5 per cent in clinical studies^29^. Results are usually given separately for R0 and R1 resections, but initially structured pathology reports were not harmonized for metastasectomies at all hospitals. Minimum resection margins were not therefore reported uniformly.

The practice presented in this study is applicable to diverse healthcare settings. Patients are not referred for treatment to the tertiary centre where the MDT recommends whether or not liver resection should be undertaken, but treated according to local practice, with the caveat that metastasectomies should be centralized to hospitals with sufficient organ-specific expertise.

*Disclosure*. The authors declare no other conflict of interest.

## Supplementary material

Supplementary material is available at *BJS* online.

## Supplementary Material

znaa145_Supplementary_Data

## Notes

Presented in part to a congress of the American Society of Clinical Oncology, Chicago, Illinois, USA, 2019, and a congress of the European Surgical Association, Cologne, Germany, May, 2020 (conference cancelled due to COVID-19); published in abstract form as *J Clin Oncol* 2019; **37**: 3517 and *Ann Surg* 2020; ESA Abstract
