# Supplementary material for: Centralized repeated resectability assessment of patients with colorectal liver metastases during first-line treatment: prospective study
Source: Br J Surg. 2021 Mar 22;108(7):817–25. doi: 10.1093/bjs/znaa145 (PMC10364914; doi:10.1093/bjs/znaa145)
Supplement: znaa145_Supplementary_Data [file znaa145_supplementary_data.zip › Isoniemi suppl List of participating 21 hospitals and investigators.docx]

**List of participating 21 hospitals and investigators**

With bold are marked the investigators fulfilling all three BJS requests for authorship.

| **Hospital** | **Investigators** |
| --- | --- |
| Helsinki University Hospital | **Pia Österlund**, **Helena Isoniemi**, **Leena-Maija Soveri, Päivi Halonen, Arno Nordin, Aki Uutela**, Riikka Huuhtanen**, Eila Lantto, Ali Ovissi**, **Juhani Kosunen**, Sirpa Leppä, Petri Bono**, Jari Räsänen**, Anna Lepistö, Heidi Penttinen, Ari Ristimäki, Olli Carpén, Nina Lundbom, Antti Hakkarainen, Marjut Timonen. |
| Turku University Hospital | **Annika Ålgars, Raija Ristamäki**, Johanna Virtanen, Eija Korkeila, Eija Sutinen, **Maija Lavonius**, Jari Sundström, Roberto Blanco |
| Tampere University Hospital | **Tapio Salminen**, Pia Österlund, Veera Salminen, Niina Paunu, Irina Rinta-Kiikka, Nieminen Lasse, **Yrjö Vaalavuo** |
| Oulu University Hospital | **Raija Kallio**, Markus Mäkinen, Eija Pääkkö **, Heikki Karjula** |
| Kuopio University Hospital | **Annamarja Lamminmäki**, Tiina Tuomisto-Huttunen, Päivi Auvinen, Vesa Kärjä, Sakari Kainulainen, Hannu-Pekka Kettunen |
| Central Finland Central Hospital | **Ilmo Kellokumpu**, Kaija Vasala, Juha Kononen, Teijo Kuopio, Kyosti Nuorva |
| Satakunta Central Hospital | Pia Österlund, Maija Murashev, Venla Viitanen, Marko Nieppola, Kalevi Pulkkanen |
| Central Hospital of Päijät-Häme | Paul Nyandoto, Aino Aalto |
| Seinäjoki Central Hospital | Timo Ala-Luhtala, Jukka Tuominiemi |
| Kymenlaakso Central Hospital | Anneli Sainast, Laura Pusa, Sanna Kosonen, Leena Helle, Timo Muhonen, Terhi Hermansson |
| Kanta-Häme Central Hospital | Riitta Kokko, Laura Aroviita, Petri Nokisalmi |
| North Karelia Central Hospital | Liisa Sailas, Heikki Tokola |
| Vaasa Central Hospital | Antti Jekunen, Teemu Pöytäkangas |
| South Karelia Central Hospital | Kari Möykkynen, Sanna Kosonen |
| Lapland Central Hospital | Olli-Pekka Isokangas, Svea Vaarala |
| South Savo Central Hospital | Rainer Kolle, Terhi Hermansson, Tuula Klaavuniemi |
| Kainuu Central Hospital | Peeter Karihtala, Mirja Heikkinen |
| Central Ostrobothnia Central Hospital | Kaisu Johansson, Anna Sjöstrand, Piia Kajasviita |
| Länsi-Pohja central hospital | Jaana Kaleva-Kerola |
| Savonlinna Central Hospital | Esa Männistö |
| Åland Central Hospital | Tom Kaunismaa, Reneé Lindvall-Andersson, Pia Vihinen, Nina Cavalli-Björkman |
