# Supplementary material for: Centralized repeated resectability assessment of patients with colorectal liver metastases during first-line treatment: prospective study
Source: Br J Surg. 2021 Mar 22;108(7):817–25. doi: 10.1093/bjs/znaa145 (PMC10364914; doi:10.1093/bjs/znaa145)
Supplement: znaa145_Supplementary_Data [file znaa145_supplementary_data.zip › Isoniemi_BJS_Suppl_Figure_1.pdf]

Supplementary Figure 1. Patient and tumour characteristics from local hospital provided online via www.raxo.fi.

R A X O

Back

Patient data

Patient number	
Patient identity number	
Hospital	Choose
Primary tumour diagnosis (Date of biopsy/operation)	(dd/mm/yyyy)
ICD-10 DG	Choose
pTNM, if operated	Date of operation (dd/mm/yyyy)
Date of diagnosis of metastasis	(dd/mm/yyyy)
Metastatic sites	☐ Liver<br>☐ Lung<br>☐ Peritoneum<br>☐ Ovary<br>☐ Bone<br>☐ Brain<br>☐ Lymph nodes<br>☐ Local recurrence:<br>☐ Other:

The following radiology will be delivered to the central radiology data base (PACS)

- ☐ MRI
- ☐ CT
- ☐ PET
- ☐ US

Date of delivery (dd/mm/yyyy)

Radiology not for immediate assessment, because

- ☐ 0-1 liver segments free of metastases
- ☐ >15 metastases in lungs
- ☐ Comorbidity inhibits operation
- ☐ Patient does not want to be operated
- ☐ Primary tumour locally inoperable / only palliative surgery possible (does not include those who are assessed to be converted to operable after treatment)
- ☐ Metastatic disease, no liver or lung metastases

Other information

Save Cancel Send
