# Supplementary material for: Centralized repeated resectability assessment of patients with colorectal liver metastases during first-line treatment: prospective study
Source: Br J Surg. 2021 Mar 22;108(7):817–25. doi: 10.1093/bjs/znaa145 (PMC10364914; doi:10.1093/bjs/znaa145)
Supplement: znaa145_Supplementary_Data [file znaa145_supplementary_data.zip › Isoniemi_BJS_Suppl_Figure_2.pdf]

Supplementary Figure 2. Repeated second opinion on resectability based on radiology in multidisciplinary team (MDT) assessment at Helsinki tertiary center provided online via www.raxo.fi to all 21 university and regional hospitals. Treatment decisions were local.

Second opinion to regional hospital

PATIENT

Data arrived — Date [ ] (dd.mm.yyyy)

Patient number [ ]

Patient identity number [ ]

Diagnosis [ ]

ICD-diagnosis [Choose]

LIVER METASTASES

Imaging
- ☐ MRI — Date of imaging [ ] (dd.mm.yyyy)
- Date [ ] (dd.mm.yyyy)
- ☐ CT — Date of imaging [ ] (dd.mm.yyyy)
- Date [ ] (dd.mm.yyyy)
- ☐ PET — Date of imaging [ ] (dd.mm.yyyy)
- ☐ US — Date of imaging [ ] (dd.mm.yyyy)

Number of metastases [ ]

Diameter of the largest metastasis [ ] mm

Localization ○ Unilateral ○ Bilateral

Segments containing metastases ☐ 1 ☐ 2 ☐ 3 ☐ 4 ☐ 5 ☐ 6 ☐ 7 ☐ 8

Resectable or not
- ○ Resectable
- ○ Not resectable now but may be convertible
- ○ Not resectable

Reason, if not resectable

All reasons
- ☐ Localization
- ☐ Number
- ☐ Size
- ☐ Extrahepatic spreading
- ☐ Other [ ]

Main reason
- ○ Localization
- ○ Number
- ○ Size
- ○ Extrahepatic spreading
- ○ Other

LUNG METASTASES

Radiological images
- ☐ MRI — Date of imaging [ ] (dd.mm.yyyy)
- Date [ ] (dd.mm.yyyy)
- ☐ CT — Date of imaging [ ] (dd.mm.yyyy)
- Date [ ] (dd.mm.yyyy)
- ☐ PET — Date of imaging [ ] (dd.mm.yyyy)
- ☐ US — Date of imaging [ ] (dd.mm.yyyy)

Number of metastases [ ]

Diameter of the largest metastasis [ ] mm

Localization ○ Unilateral ○ Bilateral

Localization in right lung
- ☐ in the upper lobe
- ☐ in the middle lobe
- ☐ in the lower lobe

Localization in left lung
- ☐ in the upper lobe
- ☐ in the lower lobe

Resectable ○ Yes ○ No

OTHER INFORMATION

Other information
Other metastatic sites recorded as absent or present, and if clearly nonresectable noted as such.
Systemic treatment recommendation and request for further radiology.
[ ]

[Save] [Cancel] [Send]
