# Supplementary material for: Centralized repeated resectability assessment of patients with colorectal liver metastases during first-line treatment: prospective study
Source: Br J Surg. 2021 Mar 22;108(7):817–25. doi: 10.1093/bjs/znaa145 (PMC10364914; doi:10.1093/bjs/znaa145)
Supplement: znaa145_Supplementary_Data [file znaa145_supplementary_data.zip › Isoniemi_BJS_Supplementary_Table1_051120.pdf]

Supplementary Table 1. Baseline characteristics of liver metastases, interventions and systemic treatments.

		**Resection or LAT**		**Systemic or BSC**	
		317	39 %	495	61 %
**Liver-limited**		208	66 %	93	19 %
**Liver & extrahepatic**		109	34 %	402	81 %
Number of metastatic sites	1	211	67 %	94	19 %
	2	76	24 %	165	33 %
	3	24	8 %	154	31 %
	4	3	1 %	63	13 %
	5-6	3	1 %	19	4 %
Number liver metastases	1	136	43 %	60	12 %
	2	66	21 %	39	8 %
	3	40	13 %	33	7 %
	4-6	38	12 %	45	9 %
	7-9	28	9 %	58	12 %
	≥10	9	3 %	260	53 %
Diameter of largest liver metastases	≤10mm	28	9 %	11	2 %
	11-20mm	89	28 %	80	16 %
	21-30mm	77	24 %	97	20 %
	31-50mm	67	21 %	110	22 %
	51-100mm	45	14 %	153	31 %
	>100 mm	11	3 %	44	9 %
Involved liver segments	1	103	32 %	44	9 %
	2	74	23 %	43	9 %
	3	61	19 %	43	9 %
	4	36	11 %	34	7 %
	5	22	7 %	43	9 %
	≥ 6	21	7 %	288	58 %
Portal vein embolization		8	3 %		
Two-stage resection		6	2 %		
RAS / BRAF mutation	RAS +/- BRAF wt	148	47 %	188	38 %
	RAS mt	151	48 %	246	50 %
	BRAF mt	9	3 %	50	10 %
	Not tested	9	3 %	11	2 %
First systemic regimen	EGFR + doublet	65	21 %	61	12 %
	VEGF + doublet/triplet	154	49 %	262	53 %
	No biologic + doublet/triplet	79	25 %	85	17 %
	VEGF + single agent	17	5 %	67	14 %
First liver resection	Conversion (+ adjuvant)	122 (76)	38 %		
	Neoadjuvant (+ adjuvant)	136 (106)	43 %		
	Adjuvant	37	12 %		
	Only resection	22	7 %		
Any ssecond resection ¶	Conversion (+ adjuvant)	31 (18)	26 %		
	Neoadjuvant (+ adjuvant)	30 (17)	25 %		
	Adjuvant	19	16 %		
	Only resection	39	33 %		

¶ number of second resection n= 119 (including 59 liver re-resections)
EGFR = Epidermal growth factor receptor inhibitor as cetuximab or panitumumab
VEGF = Vascular endothelial growth factor inhibitor as bevacizumab or aflibercept
Doublet = Fluoropyrimidine with oxaliplatin or irinotecan
Triplet = Fluoropyrimidine with oxaliplatin and irinotecan
