# Supplementary material for: Centralized repeated resectability assessment of patients with colorectal liver metastases during first-line treatment: prospective study
Source: Br J Surg. 2021 Mar 22;108(7):817–25. doi: 10.1093/bjs/znaa145 (PMC10364914; doi:10.1093/bjs/znaa145)
Supplement: znaa145_Supplementary_Data [file znaa145_supplementary_data.zip › Isoniemi_Suppl_Figure_3.pdf]

Supplementary figure 3. 12-month landmark overall survival (OS) from diagnosis of metastatic colorectal cancer (mCRC) to control guarantee-time bias.
(A) Liver-limited. (B) Liver & Extrahepatic.

A

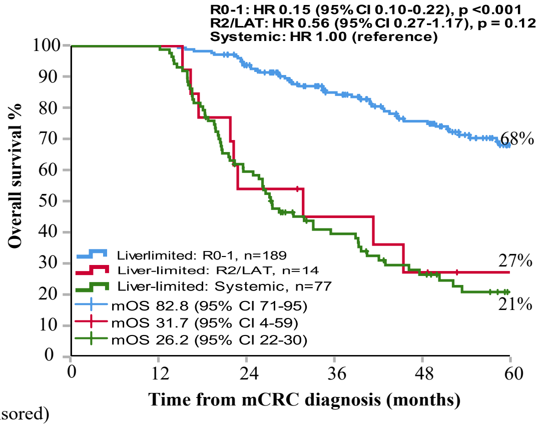

Number at risk (censored)

Liver-limited & R0-1	180 (0)	180 (14)	162 (34)	117 (51)	92 (83)	52 (124)
Liver-limited & R2/LAT	13 (0)	13 (5)	7 (1)	5 (1)	3 (3)	1 (4)
Liver-limited & Systemic	87 (0)	87 (2)	50 (8)	28 (10)	17 (17)	7 (22)

B

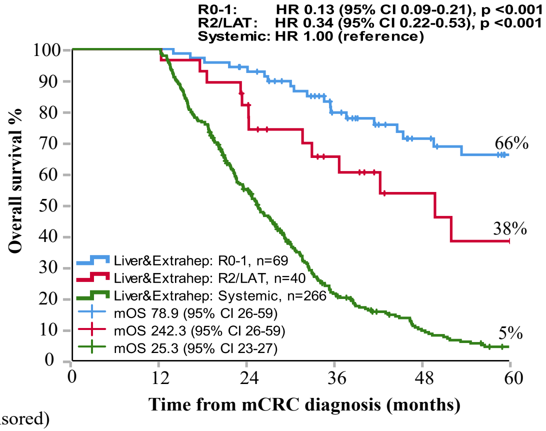

Number at risk (censored)

Liver+Extrah. & R0-1	69 (0)	69 (2)	63 (13)	44 (23)	29 (29)	21 (44)
Liver+Extrah. & R2/LAT	28 (0)	28 (1)	22 (6)	13 (10)	7 (10)	5 (12)
Liver+Extrah. & Systemic	277 (0)	277 (9)	145 (20)	51 (27)	19 (30)	6 (33)
